# Annotations

**Published:** 1911-02-11

**Authors:** 


### Annotations.

### Railway Accidents.

The recent railway accidents, which have, unfortunately, been attended with so much loss of life, may at least be confidently expected to produce one good result in drawing attention to the urgent necessity for limiting the working hours of signalmen. A continuous strain, such as that entailed by work in a signal-box or on points, is the most harassing process to which the human organism can be subjected. In the circumstances, it is scarcely to be wondered at that accidents more or less directly due to "occupation fatigue" do occur. The more wonderful thing is that they occur so rarely. At present the time during which signalmen work, in other words during which they are undergoing this wearying strain, are, in our opinion, longer than it need be. In the majority of cases the work in the box is attended with little or no relaxation; it is, for the most part, a dreary, monotonously unvarying process of pulling levers, looking at clock faces, and again pulling levers. The occupation must always—so long as we have no reliable automatic switchboards—remain a gravely responsible one; but it is sincerely to be hoped that the directors of all our railway companies will see to it that the men who follow it are not subjected to conditions which make it doubly hard for them to carry out their work. Shorter hours and more men should be the rule in the future.

### Graft and Anti-medicalism.

In that progressive land, the United States, there is much greater activity in promoting the national well-being and the national health by means of legislation than there is over here. Sometimes, it is true, individual States hurry rather too quickly in their efforts to keep in the van of progress, and mistakes are made; but on the whole it is fair to say that the public education in matters of hygiene is in advance of that in this country, as far as laws are concerned. It is, therefore, not surprising to hear that a serious effort is being made to create a national bureau or department of public health, an enterprise long needed on both sides of the Atlantic. So far, so good. It appears, however, that a "league for medical freedom" has been formed to combat the proposed bureau, and, incidentally, to discredit the medical profession at every opportunity, and in every conceivable way. One of the moves of the active organisers of the league is to procure in a well-known American magazine the insertion of an article purporting to prove that there was never a single case of plague in California, and that the assertions of the Marine Medical Service that there were such cases have been merely lies. Slips calling attention to this article have been distributed broadcast over the United States, and it is quite possible that this judicious dissemination of corrupt and tainted literature may seriously damage the cause of public health there. It is with great pleasure that we note the courageous stand made by the *Californian State Journal of Medicine*, which does not hesitate to pillory the offending magazine and this league in language so refreshingly outspoken that it must be assumed that Californian juries are less squeamish about a few hard words than British ones are, or else that there are no laws of libel in that land of oranges and Chinamen.

### Chemists' Grievances in Germany.

In Germany the shapes of medicine-bottles are regulated by law. Remedies for internal administration have to be delivered by the chemist in round bottles, on which are pasted white labels, whereas those for external use are made up in hexagonal bottles with a striated surface and labelled in red. The chemists are apparently dissatisfied at present for many reasons, and one of the causes is the return of bottles and other receptacles by their clients. Before these can be used a second time the law directs that they must be carefully cleansed and then sterilised. Moreover, all boxes, celluloid coverings, &c., must be destroyed by fire. A second cause of complaint is the vague directions for dosage given by doctors on their prescriptions. The chemists point out that in former days, when it was customary to order infusions and potions of feeble activity, it mattered little whether large or small teaspoons were made use of to measure out the medicine; but nowadays, when powerful drugs are ordered in concentrated form, the actual size of a reputed teaspoon may make all the difference between safety and danger in the matter of dosage. The true reason for this somewhat sudden love for precision is, perhaps, to be found in the increased profit which the dispensing chemist hopes to make by the sale of graduated receptacles. It cannot, however, be denied that both doctor and patient would eventually gain by more accuracy in the measurement of doses. There is yet a third grievance extant, and this is one of injured dignity. The employés in the pharmaceutical trade resent the use of terms such as *Lehrling* (apprentice) and *Gehilf* (aid or companion) as applied to their sacred selves, and lay claim to the French titles, *élève* (pupil) and *assistant* (assistant). This attitude is amusing, in view of the crusade embarked upon by purists, and in particular members of the *Deutscher Sprachverein*, for the expurgation from the German language of all terms derived from foreign sources.

### A Notice.

With reference to a paragraph in the issue of the The Hospital for December 17, 1910, entitled "An Advance Announcement," in which comments were made upon an advertisement of a new book by Mr. H. C. Ross, we desire to state that we are fully satisfied that Mr. Ross was in no way responsible for, or had knowledge of, the advertisement in question, and to withdraw unreservedly anything in the paragraph which might be calculated to give a contrary impression. We wish at the same time to express our great regret to Mr. Ross for any injury or annoyance that our comments may have caused him.

